# Colistin-Resistant *Enterobacteriaceae* Carrying the *mcr-1* Gene among Patients in Hong Kong

**DOI:** 10.3201/eid2209.160091

**Published:** 2016-09

**Authors:** Sally C.Y. Wong, Herman Tse, Jonathan H.K. Chen, Vincent C.C. Cheng, Pak-Leung Ho, Kwok-Yung Yuen

**Affiliations:** Queen Mary Hospital, Hong Kong, China (S.C.Y. Wong, J.H.K. Chen, V.C.C. Cheng);; The University of Hong Kong, Hong Kong (H. Tse, P.-L. Ho, K.-Y. Yuen)

**Keywords:** colistin, human, *Enterobacteriaceae*, antimicrobial resistance, bacteria, Gram-negative bacteria, enterobacterial infections, *Escherichia coli*, plasmids, screening, Hong Kong

**To the Editor:** Colistin belongs to the last line of bactericidal antimicrobial drugs active against multidrug-resistant gram-negative bacteria such as carbapenemase-producing *Enterobacteriaceae* and carbapenem-resistant *Acinetobacter baumannii*. Consequently, the discovery of the plasmid-mediated colistin-resistant gene *mcr-1* in *Escherichia coli* (*1*) raises concern in the medical community because colistin might be useless in treating infections caused by *mcr-1*–carrying *Enterobacteriaceae*.

During December 8, 2015–January 8, 2016, we conducted prospective laboratory surveillance of *mcr-1*–carrying *Enterobacteriaceae* and *Acinetobacter* species in a university-affiliated tertiary hospital serving a population of ≈0.53 million in Hong Kong, China. Clinical specimens were processed by using standard operating procedures for different specimen types (*2*). All *Enterobacteriaceae* and *Acinetobacter* spp. isolates were plated onto MH1 agar, which is Mueller-Hinton agar (BD Diagnostics, Sparks, MD, USA), supplemented with 1 µg/mL colistin sulfate (Sigma-Aldrich, St. Louis, MO, USA) for overnight incubation at 37°C in air. Intrinsically colistin-resistant organisms, including *Proteus* spp., *Providencia* spp., *Serratia* spp., and *Morganella* *morganii*, were excluded. *E. coli* ATCC 25922 was used as a negative control. We screened bacteria that grew on MHC1 for *mcr-1* by real-time PCR that used specific primers MCR1_22697_F1 (5′-CACTTATGGCACGGTCTATGA-3′) and MCR1_22810_R1 (5′-CCCAAACCAATGATACGCAT-3′) and the hydrolysis probe MCR1_22763_Pb1 (FAM-TGGTCTCGG/ZEN/CTTGGTCGGTCTGTAGGGC-3IABkFQ) (Integrated DNA Technologies, Coralville, IA, USA). The complete *mcr-1* gene found in PCR-positive isolates was amplified and sequenced by specific primers. The colistin MIC of positive isolates was measured by using Etest strips (BioMérieux, Marcy l’Etoile, France). Susceptibility to other antimicrobial drugs was determined by using the Kirby-Bauer disk diffusion method, according to Clinical and Laboratory Standards Institute guidelines (*3*). We retrieved clinical details of patients whose sample had *mcr-1–*carrying *Enterobacteriaceae* from the hospital clinical management system.

A total of 1,324 *Enterobacteriaceae* and 103 *Acinetobacter* spp. isolates were screened on MHC1 agar and isolated from blood, urine, stool, or respiratory samples; wound swab specimens; and other sterile and nonsterile body fluids, tissues, or swab specimens. Of the total 1,427 isolates, 62 (4.3%) grew on MHC1: 26 *E. coli*, 24 *Klebsiella* spp., 7 *Enterobacter* spp., 4 *Salmonella* spp., and 1 *Citrobacter* sp. Among these 62 isolates, 1 *Enterobacter cloaceae* and 4 *E. coli* isolates were *mcr-1* positive. All gene sequences were 100% identical to that of *mcr-1* in *E. coli* strain SHP45 (GenBank accession no. KP347127), which was isolated from a pig farm specimen in China (*1**,**3*). Of the 5 *mcr-1*–positive isolates, 2 were from blood cultures from patients with biliary tract infection, 1 from a mid-stream urine specimen from a patient with symptomatic urinary tract infection, and 2 from stool specimens from asymptomatic patients. The range of colistin MICs of the 5 *mcr-1*–positive isolates was 3–64 μg/mL; all were susceptible to carbapenem. One *E. coli* isolate (from patient 4) exhibited extended-spectrum β-lactamase activity (Table). Patient 3 resided in mainland China before this admission; patient 2 received a liver transplant in China in 2004. None of the 5 patients had a history of colistin treatment.

**Table:** Clinical details of 5 patients infected with *mcr-1*–carrying *Enterobacteriaceae*, Hong Kong*

Patient ID† (age, y)	Underlying conditions	Time from admission to collection of specimen (specimen type)	Antimicrobial drug use <1 mo before isolation	Outcome	*mcr-1*–positive species (colistin MIC, µg/mL)
1 (55)	Acute myeloid leukemia 4 mo after bone marrow transplant	4 mo (stool sample‡)	Pipericillin/ tazobactam, meropenem	Asymptomatic colonization	*Enterobacter cloacae* complex (64)
2 (68)	Primary sclerosing cholangitis with liver transplant in China in 2004; currently on sirolimus and prednisolone; right hepatectomy in 2008 for right diffuse ischemic bile injury; history of recurrent cholangitis	On admission with sepsis workup for biliary sepsis resulting from biliary anastomotic stricture (blood culture)	None	Recovered	*Escherichia coli* (3)
3 (2)	Autologous bone marrow transplant for stage IV neuroblastoma	14 d (stool sample‡)	Pipericillin/ tazobactam	Asymptomatic colonization	*E. coli* (3)
4 (57)	Hepatitis B virus–related hepatocellular carcinoma; recurrent pyogenic cholangitis; recurrent biliary sepsis with portal vein thrombosis; cerebellar stroke in 2013	On admission with sepsis workup for biliary sepsis resulting from biliary stricture and recent transarterial chemoembolization (blood culture)	None	Recovered	ESBL-producing *E. coli* (4)
5 (80)	Duke’s B carcinoma of rectum [lower anterior resection in 1996]; carcinoma of thyroid [post-thyroidectomy]; hypertension; diabetes mellitus	On admission with sepsis workup for symptomatic urinary tract infection (mid-stream urine sample)	None	Recovered	*E. coli* (4)

*ID, identifier; ESBL, extended-spectrum β-lactamase. †Patient 4 was male; others were female. ‡Routine surveillance of stool samples for multidrug-resistant organisms according to infection control protocol (*4*).

Finding the *mcr-1* gene in 0.4% of *Enterobacteriaceae* clinical isolates in Hong Kong is expected because of the high proportion of livestock and meat imported from China, where prevalence of colistin-resistant isolates is up to 25.4% and 28.0% in pigs and retail chicken meat, respectively (*5*,*6*). Our findings highlight several issues. We noted a wide range (3–64 μg/mL) of colistin MICs in the *mcr-1–*carrying *Enterobacteriaceae*; the *E. cloacae* isolate exhibited the highest MIC. This wide variation in MICs has been noted previously (*1*,*7*). Whether the variation results from the differential expression of the *mcr-1* gene or from potentially unidentified colistin resistance mechanisms co-existing in some isolates is unknown (*8*).

Our discovery of the *mcr-1* gene in an *E. cloacae* isolate adds diversity to the *Enterobacteriaceae* species known to be *mcr-1* carriers (e.g., *E. coli, Klebsiella pneumoniae,* and *Salmonella* sp.) (*1*,*9*). An in vitro study showed transfer of *mcr-1–*carrying pHNSHP45 (i.e., polymyxin-resistant plasmid) to *Pseudomonas aeruginosa* (*1*). Consequently, surveillance for the *mcr-1* gene should include all clinically relevant *Enterobacteriaceae* species, and screening for other gram-negative organisms (e.g., *P. aeruginosa*) infecting humans should be considered.

We show a potential workflow for screening *mcr-1* isolates by sequential use of MHC1 agar and real-time PCR. Clinical and Laboratory Standards Institute guidelines have no recommended colistin breakpoints for *Enterobacteriaceae* (*3*); however, the European Committee on Antimicrobial Susceptibility Testing recommends a breakpoint of >4 µg/mL to define colistin resistance in *Enterobacteriaceae* (*10*). Given that some *mcr-1*–positive isolates may have a colistin MIC of 2 µg/mL (*1*,*7*), which is lower than the recommended breakpoint, we designed MHC1 with a colistin concentration of only 1 µg/mL to minimize false-negative results. However, some colistin-susceptible organisms might grow on MHC1 (<5% in our study), resulting in the low PCR-positive rate for *mcr-1* among isolates.

Exact epidemiology of the *mcr-1* gene is unknown, indicating a need to conduct accurate surveillance of the gene’s prevalence in humans. Additional mechanisms unique to the *mcr-1* gene may contribute to colistin resistance, suggested by the wide variation in colistin MICs among *mcr-1*–carrying *Enterobacteriaceae*.
